# A Review of Redox Electrolytes for Supercapacitors

**DOI:** 10.3389/fchem.2020.00413

**Published:** 2020-06-03

**Authors:** Le Zhang, Shuhua Yang, Jie Chang, Degang Zhao, Jieqiang Wang, Chao Yang, Bingqiang Cao

**Affiliations:** ^1^Materials Center for Energy and Photoelectrochemical Conversion, School of Material Science and Engineering, University of Jinan, Jinan, China; ^2^Key Laboratory of Micro-Nano Powder and Advanced Energy Material of Anhui Higher Education Institutes, Chizhou University, Chizhou, China; ^3^School of Physics and Physical Engineering, Qufu Normal University, Qufu, China

**Keywords:** ionic conductivity, energy density, power density, redox electrolyte, supercapacitor

## Abstract

Supercapacitors (SCs) have attracted widespread attention due to their short charging/discharging time, long cycle life, and good temperature characteristics. Electrolytes have been considered as a key factor affecting the performance of SCs. They largely determine the energy density based on their decomposition voltage and the power density from their ionic conductivity. In recent years, redox electrolytes obtained a growing interest due to an additional redox activity from electrolytes, which offers an increased charge storage capacity in SCs. This article summarizes the latest progress in the research of redox electrolytes, and focuses on their properties, mechanisms, and applications based on different solvent types available. It also proposes potential solutions for how to effectively increase the energy density of the SCs while maintaining their high power and long life.

## Introduction

Supercapacitors (SCs) are a new type of energy storage equipment filling the gap between secondary batteries and traditional capacitors (Hui et al., 2019; Poonam et al., 2019; Zhi-yu et al., 2019; Alipoori et al., 2020; Cheng et al., 2020; Iqbal et al., 2020; Li and Liang, 2020; Mohd Abdah et al., 2020; Panda et al., 2020; Yi et al., 2020). SCs are believed to be one of the most promising candidates due to their fast charging/discharging capability, long cycle life, high power density, and high safety (Zhang W. et al., 2017; Zhang et al., 2018; Afif et al., 2019). In the course of decades of their development, the research on SCs has mainly focused on the preparation and modification of electrode materials to improve capacity (Zhao and Zheng, 2015). As an important part of SCs, electrolytes provide ionic conductivity and promote the charge compensation of electrodes (Wang Y. et al., 2016), so the performance of the SCs is determined by the electrolyte together with the electrode material. The electrolyte has two key parameters: (1) Electrochemical stability window. If the electrode material doesn't undergo any decomposition reaction within the voltage range of the SCs, then the output voltage of the device largely depends on the decomposition voltage of the electrolyte (Schütter et al., 2016). (2) Ionic conductivity. It affects the dynamic process and determines the rate capability of the SCs. It is related to the number of carriers, the ionic charge, and carrier mobility. The SCs electrolytes mainly have the following types: aqueous electrolytes, organic electrolytes, ionic liquid electrolytes, all solid electrolytes, gel electrolytes, and redox electrolytes (Panda et al., 2020). Several reviews concerning electrolytes for SCs have been published previously (Zhao and Zheng, 2015; Zhong et al., 2015; Pal et al., 2019; Li et al., 2020). However, none of the previous reviews concentrated on the dependence of properties, mechanisms, and applications of redox electrolytes on the different solvent types available.

Redox electrolytes are a specific type of electrolyte, in which redox active species were added. They can greatly increase the electrochemical performance of SCs for two reasons: (1) The electrolyte additive is an active part of the SCs in redox reactions during charge and discharge processes (Sankar and Selvan, 2015; Sun et al., 2015b; Fan et al., 2016; Wang C. et al., 2016). (2) The redox reactions in the electrolyte are conducive to electron transfer between the electrode material and the redox species in the electrolyte (Dai et al., 2016; Vlad et al., 2016; Gao et al., 2017; Mourad et al., 2017; Xiong et al., 2017). This review mainly summarizes the latest research results of various redox electrolytes based on aqueous, organic, ionic, and gel solvents.

## Redox Mediated Aqueous Electrolytes

Aqueous electrolytes can be divided into three types: acidic, alkaline, and neutral solutions. As a commonly used electrolyte, sulfuric acid aqueous solution not only has high ion conductivity/concentration but also low equivalent series resistance. Therefore, adding redox additives to sulfuric acid aqueous solution is a good way to optimize the electrolyte and improve the performance of SCs. Some typical redox additives contain KI (Zhang Y. et al., 2017; Gao et al., 2018), Na_2_MoO_4_ (Xu et al., 2017a), Ce_2_(SO_4_)_3_ (Díaz et al., 2015), Fe^3+^/Fe^2+^ (Ren et al., 2017), viologen substances (Sathyamoorthi et al., 2016), 1,4-dihydroxyanthraquinone (Xu et al., 2017b), hydroquinone (HQ) (Pham et al., 2015; Chen and Lin, 2019), and so on.

Generally, the energy density based on multiple redox additives is higher than that based on a single redox additive in aqueous electrolytes (Lee et al., 2016b; Teng et al., 2016). When using mixed electrolytes, their ratio is a key factor for the performance of SCs. Xu et al. (2017a) adjusted the overlapping redox voltage windows by the ratio of Na_2_MoO_4_ to KI. The optimal system (Na_2_MoO_4_: KI = 1:1) shows higher capacitance (the capacitance increased by 17.4 times) and better rate performance than other systems (Na_2_MoO_4_: KI ≠ 1:1) due to a synergistic effect between Na_2_MoO_4_ and KI. Sathyamoorthi reported on a viologen-based redox active electrolyte, in which the redox behavior of bromide and 1,1′-diethyl-4,4′-bipyridinium ions boosted both anode and cathode performance (Sathyamoorthi et al., 2016). Interestingly, the specific capacitance of the SCs increases continuously during the charge and discharge cycle, and a 30% increase is observed at the end of the 1,000 cycles. Hu et al. (2017) reported on redox additives 4-hydroxybenzoic acid (HBA), 3,4-dihydroxybenzoic acid (DHBA), and 3,4,5-trihydroxybenzoic acid (THBA) in H_2_SO_4_. SCs with HBA and DHBA exhibit higher capacitances because of their functional hydroxyl groups in the benzene ring.

For alkaline electrolytes, K_3_Fe(CN)_6_ (Veerasubramani et al., 2016; Lamiel et al., 2017) and p-phenylenediamine (PPD) (Zhang et al., 2015) can improve the capacitance and stability of the SCs. Zhang et al. (2015) introduced PPD into KOH electrolytes to form a PPD-KOH electrolyte. As expected, the specific capacitance of the carbon sample in the PPD-KOH electrolyte was larger (501.4 F g^−1^ at 3 A g^−1^) than that of SCs using an electrolyte without PPD (119.2 F g^−1^ at 3 A g^−1^). Fic et al. (2015) demonstrated a new capacitor concept in which the positive electrode works in a KI solution and the negative electrode works in a KOH electrolyte. Because of the redox reactions of I^−^/I_2_, the capacitance and energy density of the SCs is improved.

For neutral electrolytes, K_3_Fe(CN)_6_ (Lee et al., 2016a), KI (Singh and Chandra, 2016) and other additives with redox properties (Chun et al., 2015) are usually added. Chun et al. (2015) found that a high energy density of about 14 Wh kg^−1^ was obtained under methyl viologen (MV)/bromide electrolytes due to the redox reactions of Br^−^/Br${}_{3}^{-}$ and MV^2+^/MV^+^. The stability was improved by substituting heptyl viologen (HV) for MV and did not degrade after 20,000 cycles. It is believed that this electrolyte system will gain a foothold in future advanced energy storage applications.

Despite making considerable progress, the low decomposition voltage of water (1.23 V) leads to a poor energy density of SCs (Yi et al., 2020). It is also reported that the cycle performance of SCs will deteriorate after adding redox additives to the aqueous electrolytes (Chodankar et al., 2016; Singh and Chandra, 2016). This is mainly because a strong redox reaction occurs at the electrode/electrolyte interfaces, which will affect the electroactive site to a certain extent (Chodankar et al., 2016).

## Redox Mediated Organic Electrolytes

In order to increase the energy density, an organic electrolyte with a wide electrochemical stability window (around 3 V) is a good choice (Zhao and Zheng, 2015). The organic system consists of organic solvents and conductive salts. Propylene carbonate (PC) (Li et al., 2015; Salunkhe et al., 2016) and acetonitrile (AC) (Dall'Agnese et al., 2016; Jäckel et al., 2016; Singh and Chandra, 2016; Yang et al., 2017) are the most commonly used solvents in SCs. Tetraethyl ammonium tetrafluoroborate (TEABF_4_) (Li et al., 2015; Jäckel et al., 2016; Salunkhe et al., 2016; Singh and Chandra, 2016; Yang et al., 2017) and LiPF_6_ (Xie L. et al., 2016) are the most commonly used salts in SCs.

Kim et al. (2016) reported a high-performance flexible microcapacitor, which employed a poly(methyl methacrylate)-propylene carbonate-lithium perchlorate (PMMA-PC-LiClO_4_) electrolyte with hydroquinone (HQ) redox additive. The operating voltage of this system is up to 1.2 V, which is better than that of other flexible SCs under HQ-PVA-H_2_SO_4_ and PPD-PVA-KOH electrolytes (both below 1 V). The volumetric capacitance increased 35-fold due to the reversible redox reaction between hydroquinone (HQ) and benzoquinone (BQ). Also, a flexible SC with an extended operating voltage of 1.5 V, a specific capacitance of up to 363 F g^−1^, and an energy density of 27.4 Wh kg^−1^ was obtained under an organic electrolyte with ferrocene and 4-oxo-2, 2, 6, 6-tetramethylpiperidinooxy additive, due to the wide voltage of the organic electrolyte and the additional faraday capacitance from the redox mediator (Zhang et al., 2016). Dall'Agnese et al. (2016) studied the electrochemical behavior of two-dimensional titanium carbide (MXene) in acetonitrile solution with 1-ethyl-3-methylimidazolium bis(trifluoromethylsulfonyl)imide (EMITFSI) additive. The capacitance of 85 F g^−1^ was obtained at 2 mV s^−1^, while a high rate capability and good cyclability appeared. Through *in-situ* X-ray diffraction studies, it was found that EMI^+^ cations are embedded in MXene, which results in increased capacitance.

## Redox Mediated Ionic Liquid Electrolytes

Ionic liquids are generally composed of a bulky, asymmetric organic cation and a weakly coordinating inorganic/organic anion, which shows a wide electrochemical window (generally above 3.5 V), high electrochemical stability, good oxidation resistance, and so on (Brandt et al., 2013). In recent years, it was discovered that Quinones are excellent redox electrolyte additives in ionic liquid electrolytes. The introduction of hydroquinone (HQ) (Dubal et al., 2015; Sathyamoorthi et al., 2015; Xu et al., 2015) and benzoquinone (BQ) (Navalpotro et al., 2016) into the electrolyte as organic redox shuttles leads to low charge transfer resistance and contributes to the improvement of the specific capacitance and specific energy of SCs.

In addition, it is reported that the addition of tin sulfate (SnSO_4_) and vanadium sulfate (VOSO_4_) (Lee et al., 2016b) to the ionic liquid electrolyte can also significantly improve the overall performance of the SCs. Xie H. J. et al. (2016) reported two redox ionic liquids, [FcEIm][NTf_2_] and [EMIm][FcNTf], which were prepared by modifying either the [EMIm] cation or the [NTf_2_] anion with ferrocene. Based on [EMIm][FcNTf], the energy density is as high as 13.2 Wh kg^−1^, while the self-discharge at the positive electrode is fully suppressed due to the deposition of a film on the electrode. It can be seen that redox-mediated ionic liquid electrolytes are promising alternatives to conventional electrolytes. However, the problems of liquid electrolyte leakage and corrosion in liquid electrolytes have severely limited its application (Ma et al., 2015).

## Redox Mediated Gel Electrolytes

GEL is a special material between liquid and solid, which exhibits the flexibility and stability of solid and the easy diffusion of liquid (Zhi-yu et al., 2019). It has a series of advantages such as a higher ionic conductivity than solid electrolytes and good mechanical and chemical stability, etc., which makes it a promising electrolyte (Batisse and Raymundo-Piñero, 2017; Qin and Panzer, 2017; Hui et al., 2019; Li et al., 2019). Recently, a novel redox-mediated strategy for SCs was reported, which can efficiently increase the ionic conductivity and produce additional capacitance by the quick reversible redox reaction introduced by the redox mediator (Alipoori et al., 2020). The redox additives in gel polymer electrolytes usually include indigo carmine (IC) (Ma et al., 2015), 2-mercaptopyridine (PySH) (Pan et al., 2015), 1-butyl-3-methylimidazolium iodide (BMIMI) (Tu et al., 2018), alizarin red S (ARS) (Sun et al., 2016), FeBr_3_ (Wang et al., 2019), 1,4 Naphthoquinone (Hashemi et al., 2018), 1-anthraquinone sulfonic acid sodium (AQQS) (Feng et al., 2016) and 1-ethyl-3-methylimidazolium tetrafluoroborate ([EMIM]BF_4_) (Seok Jang et al., 2016).

**Figure 1:**
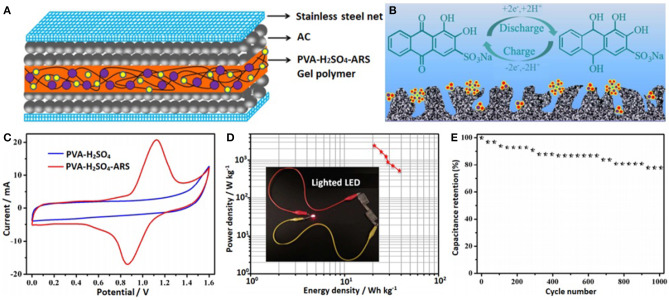
**(A)** The fabrication model of the SCs with PVA-H_2_SO_4_-ARS electrolyte, **(B)** double-layer formation and redox reaction on the carbon surface, **(C)** CV curves for the SCs at 10 mV s^−1^, **(D)** Ragone plots of the SCs with PVA-H_2_SO_4_-ARS electrolyte, **(E)** cyclic performances of the SCs with PVA-H_2_SO_4_-ARS electrolyte at 1 A g^−1^. Reproduced by permission of The Royal Society of Chemistry from Sun et al. (2016).

The redox-mediated gel polymer electrolyte (PVA-H_2_SO_4_-IC) was prepared by adding indigo carmine (IC) to a mixture of polyvinyl alcohol (PVA) and sulfuric acid (H_2_SO_4_). Its ionic conductivity is increased by 188%, reaching 20.27 mS cm^−1^. Due to the reversible redox reaction of the IC, the specific capacitance of the device was increased by 112.2% (382 F g^−1^), and the energy density also increased to 13.26 Wh kg^−1^. It also shows excellent cycling stability (80.3% capacitance retention after 3,000 cycles) (Ma et al., 2015). When alizarin red S (ARS) was added into polyvinyl alcohol-sulfuric acid (PVA-H_2_SO_4_), a new type of electrolyte (PVA-H_2_SO_4_-ARS) was obtained (Figure 1). Its conductivity reached 33.3 mS cm^−1^, due to ARS acting as a redox shuttle in the electrolyte. Compared with ARS-free SCs (160 F g^−1^ at 0.5 A g^−1^), the specific capacitance of SCs using a PVA-H_2_SO_4_-ARS gel polymer electrolyte is larger (441 F g^−1^ at 0.5 A g^−1^). At the same time, its energy density is as high as 39.4 Wh kg^−1^ and it has a good cycling stability. Therefore, the redox-mediated electrolyte has a good application prospect in improving the electrochemical performance of SCs (Sun et al., 2016).

Sun et al. (2015a) prepared a redox-mediated gel polymer-polyvinyl alcohol-orthophosphate 2-mercaptopyridine (PVA-H_3_PO_4_-PySH) by introducing PySH into PVA-H_3_PO_4_. The ionic conductivity of the PVA-H_3_PO_4_-PySH system was increased by 92% to 22.57 mS cm^−1^. As a result, a high specific capacitance (1,128 F g^−1^) and energy density (39.17 Wh kg^−1^) were obtained. These improved properties are attributed to the redox reaction between PySH and 2,2′-bipyridine redox couple in PVA-H_3_PO_4_-PySH (Ye et al., 2018). These results undoubtedly indicate that redox-mediated gel polymers are promising electrolyte candidates for advanced flexible SCs (Aljafari et al., 2019).

Although redox electrolytes have greatly contributed to the improvement of the performance of SCs, it's worth noting that self-discharge (SD) is a fatal weakness for most redox electrolytes. So, many pieces of research have focused on this problem recently. Fan et al. (2020) lowered self-discharge and improved energy density and cycling stability (capacitance retention 87.9% after 10,000 cycles) by the addition of Li_2_SO_4_-BMIMBr-carbon nanotubes in the PVA solution, in which the 3D carbon nanotubes networks provide fast ion transmission channels. Chen et al. (2014) blocked the migration of the active electrolyte between two electrodes and suppressed the self-discharge though inhibiting BQ shuttle with Nafion®177 membrane or suppressing shuffle effect with a CuSO_4_ active electrolyte. It is believed that these results will guide the further design of SCs with both a high energy density and good energy retention.

In the end, a table (Table 1) was given, in which several typical redox electrolyte-based SCs are summarized and compared for clarity.

**Table 1:** Redox electrolyte-based SCs and their performance (Mai et al., 2013; Park et al., 2014; Yu et al., 2014; Díaz et al., 2015; Sathyamoorthi et al., 2015; Zhang et al., 2015; Kim et al., 2016; Navalpotro et al., 2016; Seok Jang et al., 2016; Singh and Chandra, 2016; Xie H. J. et al., 2016; Mousavi et al., 2017; Ren et al., 2017; Gao et al., 2018; Tu et al., 2018; Wang et al., 2019).

	**Redox additives**	**Supporting electrolyte**	**Capacitance**	**Energy density (Wh kg^**−1**^)**	**Cycling stability**
Redox mediated aqueous electrolytes	KI	H_2_SO_4_	203–616 F g^−1^ at 1 A g^−1^	—	77.3% capacitance retention after 5,000 cycles
	Ce_2_(SO_4_)_3_	H_2_SO_4_	408 F g^−1^ at 17.7 mA cm^−2^	1.24–13.84	94% capacitance retention after 3,000 cycles
	0.8 M Fe^3+^/Fe^2+^	H_2_SO_4_	1,062 F g^−1^ at 2 A g^−1^ (almost tripled)	8.3–22.1	93% capacitance retention after 10,000 cycles
	Catechol	H_2_SO_4_	429–1,967 F g^−1^ at 1 A g^−1^	81.8	80% capacitance retention after 5,000 cycles
	CuCl_2_	HNO_3_	440–4,700 F g^−1^ at 5 mVs^−1^	163	99.4% capacitance retention after 5,000 cycles
	PPD	KOH	119.2–501.4 F g^−1^ at 3 A g^−1^	—	85.2% capacitance retention after 5,000 cycles
	KI	Li_2_SO_4_	96–198 F g^−1^ at 1 A g^−1^	65	85.3% capacitance retention after 3,000 cycles
Redox mediated organic electrolytes	HQ	PMMA	0.2–7.1 mF cm^−2^ at 0.1 mA cm^−2^	—	97% capacitance retention after 10,000 cycles
	PPD	LiClO_4_+AC	25–69 F g^−1^ at 0.5 A g^−1^	18–54	93% capacitance retention after 5,000 cycles
	DmFc	TBAP+THF	8.3–61.3 F g^−1^ at 10 A g^−1^	36.8	88.4% capacitance retention after 10,000 cycles
Redox mediated ionic electrolytes	HQ	TEATFSI	72 F g^−1^ at 0.57 mA cm^−2^	18.4–31.22	84.1% capacitance retention after 1,000 cycles
	p-BQ	PYR_14_TFSI	20–70 F g^−1^ at 5 mA cm^−2^	3.5–10.3	50% capacitance retention after 1,000 cycles
	Ferrocene	[EMIM] [NTf_2_]	—	7.2–13.2	—
Redox mediated gel electrolytes	FeBr_3_	H_2_SO_4_+PVA	204–885 F g^−1^	33.9	100% capacitance retention after 10,000 cycles
	BMIMI	Li_2_SO_4_+PVA	139.1–384 F g^−1^ at 0.25 A g^−1^	10.4–29.3	80.9% capacitance retention after 10,000 cycles
	[EMIM]BF_4_	H_3_PO_4_+ PVA	103–271 F g^−1^ at 0.5 A g^−1^	20.7–54.3	70% capacitance retention after 3,000 cycles

*EVD, ethyl viologen dibromide; AQDS, anthraquinone-2,7-disulphonate; PMMA, poly (methyl methacrylate); DmFc, decamethylferrocene; TBAP, tetrabutylammonium perchlorate; THF, tetrahydrofuran; TEATFSI, triethylammonium bis(trifluoromethane)sulfonamide; p-BQ, para-benzoquinone; PYR_14_TFSI, N-butyl-N-methyl pyrro lidinium bis(trifluoromethanesulfonyl) imide; [EMIM][NTf_2_], 1-ethyl-3-methylimidazolium bis(trifluoromethanesulfonyl)imide*.

## Conclusions and Perspectives

Each redox electrolyte has its own advantages and disadvantages. The two most important criteria for selecting an electrolyte are the operating voltage and the ionic conductivity. The higher the operating voltage and the ionic conductivity is, the greater the energy density and power density of the SCs. In order to further develop high-performance SCs electrolytes and improve the overall performance of SCs, we can start from the following aspects: (1) The introduction of redox active materials that can produce reversible redox reactions in the electrolyte is an effective way to increase the capacity and energy density of SCs; (2) Investigating the interaction mechanism between the electrode material and the electrolyte, and optimizing the matching relationship between them. Taken together, these redox electrolytes pave the way for high-performance SCs applications.

## Author Contributions

All authors listed have made a substantial, direct and intellectual contribution to the work, and approved it for publication.

## Conflict of Interest

The authors declare that the research was conducted in the absence of any commercial or financial relationships that could be construed as a potential conflict of interest.
